# Intranasal Drug Delivery Technology in the Treatment of Central Nervous System Diseases: Challenges, Advances, and Future Research Directions

**DOI:** 10.3390/pharmaceutics17060775

**Published:** 2025-06-13

**Authors:** Xunxun Wu, Ranqing Zang, Yiting Qiu, Yufang Zhang, Junbin Peng, Zhiyun Cheng, Site Wei, Meiyan Liu, Yong Diao

**Affiliations:** School of Medicine, Huaqiao University, Quanzhou 362021, China; wuxunxun2015@hqu.edu.cn (X.W.); 13612820860@163.com (R.Z.); qytdezh@163.com (Y.Q.); zhangyufang@stu.hqu.edu.cn (Y.Z.); pengjunbin@stu.hqu.edu.cn (J.P.); chengzhiyun@hqu.edu.cn (Z.C.); wei5826013@163.com (S.W.); 15863607363@163.com (M.L.)

**Keywords:** intranasal drug delivery, central nervous system, blood–brain barrier, olfactory nerve pathway, trigeminal nerve pathway, nanotechnology, hydrogels, Alzheimer’s disease, small molecules

## Abstract

As population aging becomes an increasingly critical global issue, the incidence of central nervous system (CNS) diseases, including Alzheimer’s disease (AD), Parkinson’s disease (PD), and stroke, has risen sharply. However, the blood–brain barrier (BBB) presents a significant obstacle to the effective treatment of these CNS disorders, limiting the ability of therapeutic agents to reach the brain. In this context, intranasal drug delivery, which bypasses the BBB, has attracted considerable attention in recent years. By utilizing pathways such as the olfactory and trigeminal nerves, intranasal drug delivery facilitates the rapid transport of drugs to the brain, thereby enhancing both the bioavailability and targeting efficiency of the drugs. This review provides an overview of the molecular mechanisms underlying intranasal drug delivery, its advancements in the treatment of CNS diseases, strategies to improve delivery efficiency, and a discussion of the challenges and potential future directions in this field. The aim of this paper is to offer valuable insights and guidance for researchers and clinicians working in the area of CNS disease treatment.

## 1. Introduction

Crossing the blood–brain barrier (BBB) has long been a significant challenge in the development of novel therapeutics for central nervous system (CNS) diseases [1]. Traditional CNS drug delivery approaches, including oral and intravenous administration, are hindered by several critical limitations. The BBB severely restricts drug penetration into the central nervous system, while metabolic degradation and first-pass hepatic effects reduce systemic bioavailability. Non-specific distribution often leads to off-target toxicity. Additionally, poor CNS permeability, short half-life, narrow therapeutic windows, and frequent dosing requirements further limit clinical efficacy and patient adherence. These challenges underscore the necessity for more efficient and targeted CNS drug delivery systems. Unlike traditional oral or intravenous routes, intranasal drug delivery facilitates drug absorption through the nasal mucosa, allowing direct access to systemic circulation and avoiding the first-pass metabolism in the liver and gastrointestinal tract, and offers a promising solution for bypassing the BBB. This enhances the bioavailability of drugs [2,3].

Consequently, identifying strategies to bypass the BBB has become a key area of scientific inquiry. Intranasal drug delivery represents a promising, non-invasive approach that circumvents the BBB, enabling direct drug delivery to the brain [4,5,6]. The nasal mucosa is rich in blood vessels and microvilli, significantly increasing the surface area for drug absorption and providing an efficient pathway for drugs to enter the bloodstream [7,8,9]. Furthermore, the olfactory epithelium, connected to various brain regions via the olfactory and trigeminal nerves, serves as a crucial route for drug transport to the brain. This connection not only enables targeted delivery to specific brain regions but also allows for precision treatment of various neurological diseases [4,10,11]. The non-invasive nature of intranasal drug delivery alleviates the drawbacks associated with invasive procedures such as injections or surgery, thus improving patient compliance—especially in the case of chronic diseases like neurodegenerative disorders, which require long-term treatment. Additionally, intranasal delivery bypasses the first-pass metabolism in the liver, thereby reducing systemic drug exposure and decreasing the potential for systemic side effects [7,12,13]. This makes intranasal drug delivery particularly advantageous for drugs with low bioavailability or poor stability [14]. In recent years, this method has gained considerable attention in the treatment of CNS disorders and beyond [15,16,17]. Numerous studies have now progressed to clinical trials, rigorously evaluating safety, efficacy, and dosing to bridge preclinical findings with real-world therapeutic applications. For example, in a phase 2/3 clinical trial, intranasal administration of zavegepant for the treatment of migraine has achieved positive results, significantly improving patients’ pain freedom [18]. In addition, the FDA has approved the use of intranasal administration of Foralumab for the first patient with moderate Alzheimer’s disease. Meanwhile, nalmefene nasal spray has been approved for the emergency treatment of known or suspected opioid overdose [19,20]. Another clinical study has explored the esketamine nasal spray combined with a selective serotonin reuptake inhibitor or a serotonin-norepinephrine reuptake inhibitor significantly improved remission and reduced relapse in treatment-resistant depression compared to quetiapine augmentation [21].

This paper provides a comprehensive review of the molecular mechanisms underlying intranasal drug delivery, its advancements in treating CNS diseases, and strategies to enhance its delivery efficiency. It also examines the challenges faced by intranasal delivery, including issues related to unstable drug absorption, local irritation, and the development of drug formulations. Finally, the paper discusses the future directions of intranasal drug delivery systems and proposes strategies for improving drug permeability, stability, and personalized treatment. This review synthesizes the latest research findings and technological innovations in the field, offering valuable insights for researchers and clinicians.

## 2. Molecular Mechanisms of Intranasal Drug Delivery

**Figure 1:**
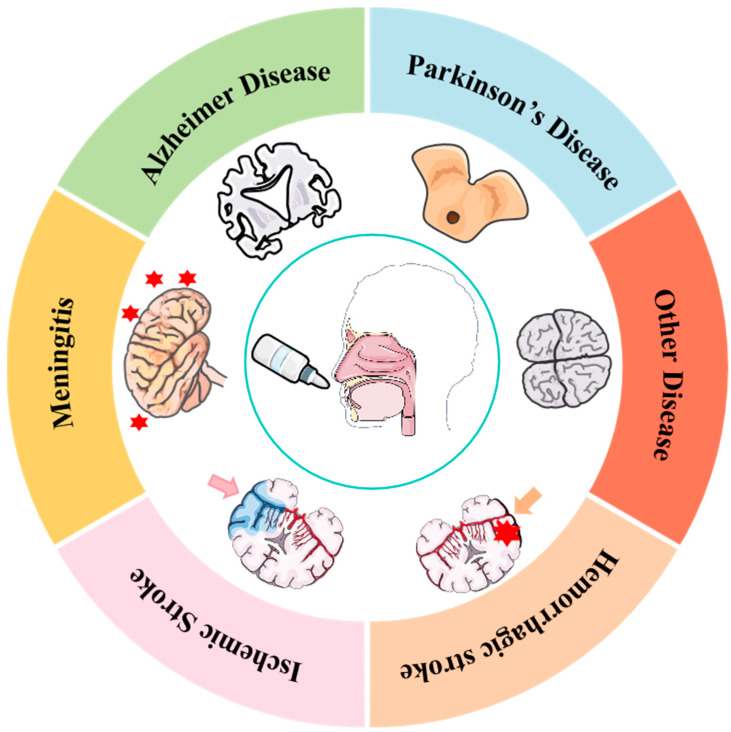
This illustration shows various central nervous system diseases that can be treated via intranasal drug delivery, including Alzheimer’s disease, Parkinson’s disease, meningitis, ischemic stroke, hemorrhagic stroke, and other unspecified diseases. The areas marked by red stars or indicated by arrows represent the sites of tissue damage or functional loss. (all or parts of the figures were created using Servier Medical Art (https://smart.servier.com/), accessed on 5 April 2025, licensed under CC BY 4.0.).

Intranasal drug delivery primarily relies on the olfactory and trigeminal nerves, utilizing both intracellular and extracellular transport pathways to enable direct access to the CNS [22]. Drugs administered via the olfactory route cross the cribriform plate to reach the olfactory bulb and other brain regions, while those delivered through the trigeminal nerve are transported to the brainstem via the anterior lacerated foramen [23]. Intracellular transport involves endocytosis by olfactory sensory neurons followed by axonal transport, whereas extracellular transport enables paracellular diffusion across the nasal epithelium into the perineural space, eventually reaching the subarachnoid space via cerebrospinal fluid (CSF) flow. The CSF, driven by arterial pulsations, distributes the drug throughout the brain via the glymphatic system, facilitating brain-wide diffusion [24,25,26] (Figure 1).

### 2.1. Olfactory Nerve Pathway

**Figure 2:**
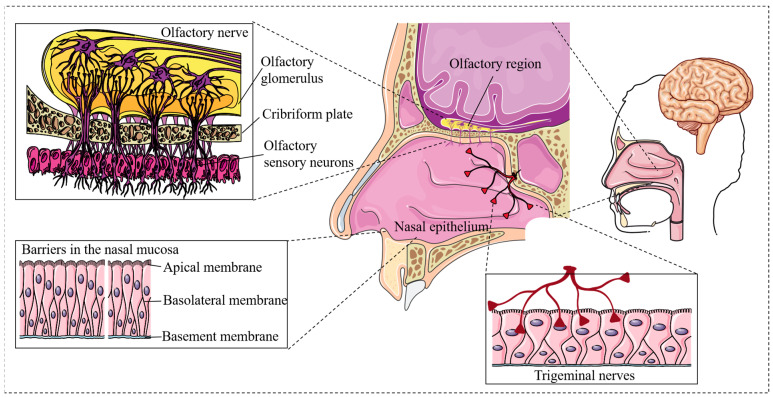
This illustration depicts the mechanisms of intranasal drug delivery. The therapeutics can be transported from the nose to the brain through the olfactory and trigeminal nerves and vascular transport across the BBB. The primary routes for nose-to-brain transport are olfactory and trigeminal nerve pathways (all or parts of the figures were created using Servier Medical Art (https://smart.servier.com/), accessed on 5 April 2025, licensed under CC BY 4.0.).

The olfactory nerve pathway is the most extensively studied mechanism for intranasal drug delivery to the brain. Drugs absorbed by the olfactory epithelium are transported along olfactory nerve fibers, bypassing the BBB and directly reaching the cerebral cortex, hippocampus, and other brain regions. This provides a crucial foundation for the treatment of CNS diseases such as stroke, epilepsy, and traumatic brain injury [8,9]. The olfactory pathway supports the transport of not only small molecules but also peptides, proteins, and macromolecules that are otherwise restricted by the BBB, achieving rapid absorption and high brain concentrations [4,27] (Figure 2).

Upon entering the olfactory mucosa, drugs follow two primary routes to reach the CNS: (1) Intracellular transport, wherein drugs undergo clathrin-mediated endocytosis by olfactory receptor neurons, followed by microtubule-based axonal transport to the olfactory bulb, where they are released into the cytoplasm. This process is relatively slow, typically requiring hours to days. (2) Extracellular transport, which is faster and involves passive transcellular or paracellular diffusion through epithelial tight junctions or membranes into the extracellular space of olfactory nerve bundles. From there, drugs are carried by bulk flow to the olfactory bulb and diffuse into the CSF, enabling widespread brain distribution [22,23,28]. Nanoparticles exhibit enhanced diffusion along this route due to their affinity for the lipid-rich environment, while hydrophilic compounds may require protein carriers. Nanoparticles with sizes ranging from 50 to 200 nm, particularly those under 150 nm, are most suitable for nose-to-brain delivery. Liposomes with a particle size below 150 nm have demonstrated enhanced brain deposition and prolonged release (up to 96 h), significantly improving drug bioavailability compared to conventional formulations [29,30]. Nanoemulsions typically sized between 20 and 200 nm (e.g., 106.8 nm) have shown a 1.68-fold increase in brain permeation efficiency [31]. Solid lipid nanoparticles, often within the 100–300 nm range, with optimized sizes around 129 nm, allow for sustained drug release and higher brain targeting [32]. Similarly, nanostructured lipid carriers around 190.98 nm have achieved superior brain delivery efficacy, with intranasal administration yielding higher AUC values than intravenous routes. Overall, particles within this nanometric range ensure effective mucosal adhesion, enhanced permeability through olfactory and trigeminal pathways, and minimized systemic side effects, making them ideal for central nervous system targeting via nasal administration [33]. These unique anatomical and molecular features render the olfactory pathway a key route for intranasal CNS drug delivery.

### 2.2. Trigeminal Nerve Pathway

In addition to the olfactory route, the trigeminal nerve serves as a significant non-invasive conduit for direct drug transport to the brain. The trigeminal nerve regulates facial sensory function and projects to the brainstem and thalamic regions [34,35,36] (Figure 2).

Drugs administered through the nasal mucosa are absorbed by epithelial cells and interact with sensory neurons of the trigeminal nerve, facilitating their delivery to deeper CNS structures. This pathway may involve activation of transient receptor potential (TRP) channels, such as TRPV1, which sensitize nerve endings and mediate receptor-dependent endocytosis, followed by dynein-driven retrograde axonal transport to brainstem nuclei [37,38]. The maxillary and ophthalmic branches of the trigeminal nerve innervate the nasal respiratory mucosa and terminate in the pons and cribriform plate. Drug transport via the trigeminal pathway may occur through both intra-axonal transport and extracellular diffusion, operating independently of the olfactory route [24]. One major distinction lies in the target site: while the olfactory pathway delivers drugs to the olfactory bulb, the trigeminal route carries them to the pons, resulting in slower diffusion into the CSF due to the longer anatomical distance to CNS targets [39,40]. Although both pathways have been validated for brain delivery, the relative contribution of each remains to be elucidated. The trigeminal nerve, as the fifth cranial nerve composed of ophthalmic, maxillary, and mandibular branches converging at the trigeminal ganglion, originates from the pons and provides a viable structural basis for CNS-targeted delivery. Drugs absorbed via the ophthalmic and maxillary branches can reach the brainstem and ultimately the brain parenchyma. Similar to the olfactory route, the trigeminal nerve supports nose-to-brain transport through both intra-axonal and extracellular mechanisms, although intracellular transport along this pathway tends to be slower [28,41,42]. These anatomical and physiological differences underscore the importance of further studies to quantify the relative contributions of each pathway and optimize intranasal formulations accordingly.

### 2.3. Transduction Mechanisms of Paracellular and Transcellular Pathways in Intranasal Drug Delivery

Beyond the neural pathways, drugs may also traverse the neurovascular spaces surrounding the olfactory and trigeminal nerves. These interstitial fluid-filled compartments enable both paracellular and transcellular diffusion into the CNS, providing an additional mechanism to enhance intranasal drug delivery efficiency [43,44]. This supplementary route facilitates drug penetration into deeper brain structures, complementing neural transport [4,35,45,46] (Figure 2).

Paracellular transport allows passive drug diffusion between epithelial cells, entering the perineural space and subsequently the subarachnoid space via CSF. Within the CSF, arterial pulsations drive distribution through glymphatic (perivascular) pathways, functioning similarly to a brain-specific lymphatic system and ensuring widespread drug delivery [10,47]. Tight junction integrity, maintained by structural proteins such as claudin-4, claudin-5, occludin, and zonula occludens-1 (ZO-1), plays a critical role in regulating this transport route [35,48]. Notably, insulin and interferon-*β* have demonstrated effective CNS targeting via the paracellular route, diffusing across epithelial junctions into the CSF [49,50].

Transcellular transport encompasses multiple mechanisms, including clathrin-mediated endocytosis, caveolae-mediated endocytosis, macropinocytosis, carrier-mediated active transport, and efflux systems. Small molecules, such as peptides and nanoparticles, commonly enter cells via clathrin-coated vesicles involving clathrin heavy chain and adaptor protein complex 2. Lipophilic agents like paclitaxel in liposomes are predominantly absorbed via caveolae-mediated pathways, while macromolecular drugs, including exosomes and proteins, are mainly internalized through micropinocytosis [49,50].

## 3. Advances in Intranasal Drug Delivery Applications

**Figure 3:**
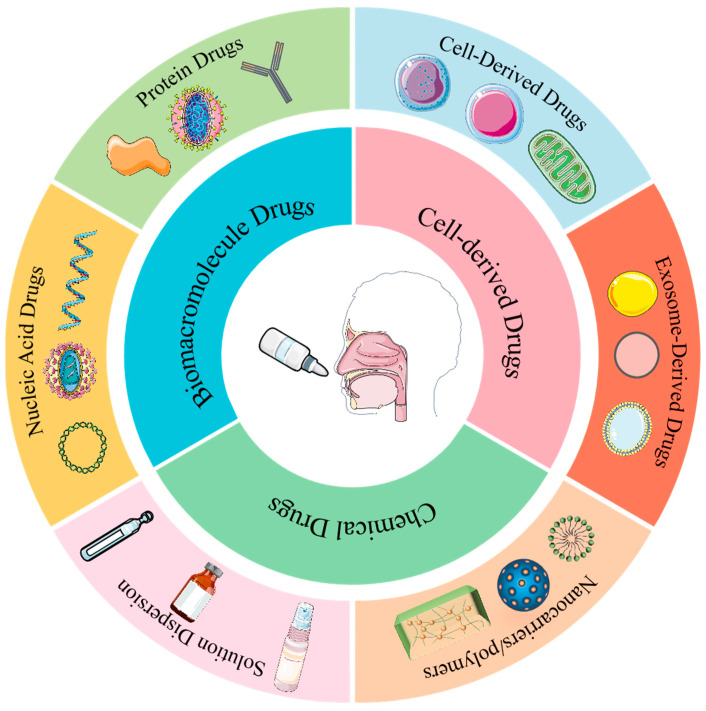
The diagram illustrates different types of drugs that can be delivered via the intranasal route, including exosome-derived drugs, cell-derived drugs, drug delivery systems, chemical drugs (in the form of solutions or dispersions), biological drugs, and nucleic acid drugs (all or parts of the figures were created using Servier Medical Art (https://smart.servier.com/), accessed on 5 April 2025, licensed under CC BY 4.0.).

Intranasal drug delivery demonstrates broad therapeutic potential, encompassing diverse agents such as chemical drugs, biomacromolecule drugs, and cell-derived drugs. As a non-invasive alternative to injections or oral delivery, it enhances patient compliance while enabling targeted therapeutic effects (Figure 3, Table 1).

### 3.1. Progress in Intranasal Delivery of Small Molecule Drugs

Small molecule drugs are widely used in clinical practice due to their small molecular weight and good oral absorption. Intranasal delivery of small molecule drugs has been widely explored, particularly in the treatment of CNS diseases. For example, intranasal delivery of galantamine, 9-cis retinoic acid, lacosamide, or isocyanomethane has shown effectiveness in treating AD and epilepsy [51,52,53,54]. Furthermore, intranasal delivery has also achieved significant results in pain management, sedation, and smoking cessation therapy, such as intranasal ketamine, dexmedetomidine, lidocaine, and chloral hydrate, which effectively alleviate postoperative pain and sedation [55,56,57,58,59]. Intranasal delivery offers advantages such as rapid onset, ease of use, and good patient tolerance, especially for advanced patients who have difficulty swallowing oral drugs [60]. In palliative care and community hospice treatment, intranasal delivery of morphine or midazolam can effectively treat pain and agitation, with simple administration and excellent suitability for patients with swallowing difficulties or fear of needles [61]. Additionally, intranasal drug delivery technology can be combined with nanotechnology or biomaterials to further enhance drug penetration and immune response, providing new delivery strategies for early intervention in neurodegenerative diseases such as AD [62,63,64] (Table 2). The above research provides a non-invasive, convenient, and efficient therapeutic approach for the intranasal delivery of small molecular chemical drugs.

### 3.2. Research Progress of Intranasal Delivery of Biomacromolecule Drugs

Biomacromolecule drugs (such as proteins, antibodies, vaccines, etc.) typically face challenges such as low bioavailability and difficulty penetrating biological barriers due to their large molecular size and complex structures [66]. These challenges often make the delivery of biomacromolecule drugs more complex than that of traditional small-molecule drugs. Therefore, the delivery of biomacromolecule drugs has been a key challenge limiting their clinical application. However, intranasal drug delivery offers significant advantages for the delivery of biologic drugs.

#### 3.2.1. Progress in Intranasal Delivery of Growth Factors and Peptide Drugs

Intranasal drug delivery offers a key advantage by bypassing the gastrointestinal tract and first-pass metabolism, thereby significantly improving the delivery efficiency of growth factors and peptide drugs. However, its effectiveness is still limited by factors such as the molecular characteristics of the growth factor, intranasal absorption capacity, stability, and the local environment. For example, intranasal delivery of nerve growth factor (NGF) has shown good therapeutic potential in treating traumatic brain injury (TBI) and other neurodegenerative diseases such as AD and PD, providing new insights for the clinical application of NGF. However, there are also some side effect risks associated with intranasal delivery, so future research needs to focus on areas such as dose optimization, combination therapy, and improvements in delivery technology to further enhance the efficacy and local drug accessibility of the drugs [67,68,69,70]. Additionally, intranasal delivery of leukemia inhibitory factor (LIF) has provided a new approach for treating neurological function after mild TBI in children [71].

Intranasal delivery is also widely applied in the delivery of peptide drugs. Intranasal delivery of insulin can enter the brain through the olfactory or trigeminal nerves, improving cognitive function with good safety [72]. Using nanotechnology to further enhance insulin delivery efficiency can also improve its therapeutic effect [73,74,75]. Clinical trials and animal studies have shown that intranasal insulin delivery improves memory performance and has significant neuroprotective effects related to diabetes [76,77,78,79,80,81]. Another notable example is the MemAID trial, a Phase II randomized controlled study involving older adults with and without type 2 diabetes. Daily intranasal administration of 40 IU insulin over 24 weeks led to significant improvements in gait speed, prefrontal cerebral blood flow, and insulin sensitivity in diabetic patients, while non-diabetic participants showed enhanced executive function and verbal memory [82]. Furthermore, the intranasal route has provided new opportunities for delivering glucagon-like peptide-1 (GLP-1) to the brain for the treatment of obesity [83]. The novel oxytocin intranasal spray TTA-121 may achieve effective treatment with lower doses and fewer sprays, offering new hope for the treatment of autism spectrum disorder [84,85]. In addition, a randomized controlled trial investigated the efficacy of intranasal oxytocin as an adjunct to exposure therapy for social anxiety disorder (SAD). The study demonstrated that oxytocin administration improved positive evaluations of appearance and speech performance during exposure sessions, indicating its potential to enhance treatment outcomes for SAD [86]. Intranasal delivery of the bicyclic peptide OL-CTOP, which contains two disulfide bonds, can effectively antagonize the analgesic effect of morphine and prevent its respiratory suppression side effects, demonstrating its potential as a novel brain-targeting drug [87] (Table 3).

#### 3.2.2. Research Progress in Intranasal Vaccine Delivery

Intranasal vaccines can deliver antigens directly to CNS-associated lymphoid tissues and neural structures. This allows for neuroimmune modulation, which is particularly valuable in treating or preventing neuroinflammatory and neurodegenerative diseases such as Alzheimer’s disease, Parkinson’s disease, and multiple sclerosis [88,89]. Previous studies have demonstrated that viruses like the hepatitis virus can enter the brain from the nasal cavity through the olfactory nerve, bypassing the BBB [90]. In addition, HHV-6 has been implicated in neurological conditions such as multiple sclerosis and encephalitis through nasal-brain transmission [91]. Experimental studies with adeno-associated virus (AAV) vectors have also shown that gene therapies can reach the brain through intranasal administration and exert therapeutic effects [92,93,94]. These findings underscore the nasal cavity as both a potential route for viral neuroinvasion and a promising pathway for non-invasive therapeutic delivery. Intranasal mucosal vaccines can effectively activate the mucosal immune system and overcome the limitations of traditional vaccines, addressing issues related to immunogenicity, stability, and safety that traditional vaccines face [95,96,97]. This is especially important for the prevention of respiratory diseases [15,87,96]. Additionally, intranasal delivery of subunit vaccines and nanomaterials can trigger both local and systemic immune responses, with breakthroughs particularly in penetrating the intranasal epithelial barrier [95,98,99]. Studies on vaccines targeting various pathogens such as the novel coronavirus (SARS-CoV-2), pneumococcus, and tuberculosis show that intranasal vaccines offer advantages of higher compliance, lower costs, and the ability to induce strong immune responses, making them especially suitable for large-scale immunization campaigns and more easily accepted by children [100,101,102]. Furthermore, intranasal vaccination is more effective than subcutaneous vaccination in alleviating clinical symptoms and pulmonary lesions, indicating that intranasal vaccination has advantages during epidemic outbreaks [103]. Intranasal vaccine delivery strategies provide new research directions for the prevention and control of respiratory viral infections [104,105,106].

Intranasal delivery of receptor-binding domain nanoparticles can effectively activate local immune responses and cellular immune responses. This provides a theoretical basis for the development of COVID-19 vaccines [107,108]. Intranasal immunization with nicotine vaccine candidates can induce both systemic and mucosal antibodies, which specifically neutralize nicotine, offering the potential for developing new smoking cessation therapies [109]. Additionally, adjuvants such as xanthan gum can enhance T cell responses and provide effective protection, while monoclonal antibody immune complexes can induce long-lasting immune responses [110,111]. Overall, these advancements offer new directions and technological support for the development of better intranasal vaccines.

#### 3.2.3. Research Progress in Intranasal Delivery of Nucleic Acid Drugs

Current gene therapy applications in the brain are limited by existing delivery systems. Intranasal delivery of CRISPR-dCas9 system-based nanoparticles has effectively upregulated the expression of the target gene Sirt1, reducing brain edema and improving survival rates after permanent middle cerebral artery occlusion [112]. Intranasal delivery of self-assembled antagomir-21/RAP nanoparticles can enhance glioblastoma treatment effects without using potentially toxic carriers [113]. Intranasal delivery of apolipoprotein A-I nanoparticles carrying antisense oligonucleotide (ASO) can reduce mutated Huntington protein levels in a Huntington’s disease mouse model [114]. Hyaluronidase-coated glycerol chitosan-DNA complexes enhance gene targeting and distribution in the brain, providing a new non-invasive gene therapy approach for neurodegenerative diseases such as AD [115]. Intranasal delivery of nucleic acid drugs is a non-invasive, fast, cell-free, and targeted therapeutic method that can significantly promote functional recovery after complete spinal cord injury (SCI) [116] (Table 4). Preclinical studies have demonstrated the efficacy of this delivery method. For instance, intranasal administration of siRNA-loaded lipid nanoparticles has shown significant reductions in glioma growth in rodent models. Similarly, intranasal delivery of mRNA vaccines has led to antigen expression in olfactory bulb neurons, indicating successful CNS targeting [117,118,119]. Intranasal delivery of nucleic acids offers a promising non-invasive route to the CNS. It effectively bypasses the BBB. However, challenges such as susceptibility to nuclease degradation and limited membrane permeability necessitate the use of protective carriers to ensure stability and facilitate cellular uptake. Once administered intranasally, nucleic acid can access the brain via two primary neuronal pathways: the olfactory and trigeminal nerves. The olfactory pathway allows direct axonal transport from the nasal epithelium to the olfactory bulb, while the trigeminal pathway provides access to the brainstem. Additionally, astrocytes and microglia can internalize these carriers through endocytosis or receptor-mediated mechanisms, including interactions with scavenger receptors [43,44]. These findings underscore the potential of intranasal nucleic acid delivery systems in treating various CNS disorders.

### 3.3. Research Progress in Intranasal Delivery of Cell-Derived Therapeutic Drugs

#### 3.3.1. Research Progress in Intranasal Delivery of Cell Therapy Drugs

In recent years, this approach has made significant progress in the delivery of biologic drugs, particularly cell-based therapies. This delivery route leverages the direct pathway between the intranasal cavity and the CNS, providing a new strategy for the treatment of CNS diseases. For example, intranasal delivery of human olfactory mucosal progenitor cells (OMPCs) or human neural stem cells (hNSCs) can specifically migrate to the vicinity of damaged neurons and axons, offering a non-invasive stem cell therapy for brain injury with potential clinical application value [120,121]. Intranasal delivery of hNSCs into the brain, where they differentiate into neurons, reduces β-amyloid plaque accumulation, decreases neuroinflammation, and improves cognitive function, providing a new avenue for the treatment of AD [122]. Additionally, intranasal delivery of bone marrow-derived mesenchymal stem cells (BMSCs) or deciduous dental pulp stem cells (DPSCs) significantly improves motor coordination and olfactory function in PD mice, reducing the degeneration of dopaminergic neurons and offering new methods for PD treatment [123,124]. Intranasal delivery of mesenchymal stromal cells (MSCs) can also treat neuronal damage caused by prion diseases [125,126], and intranasal delivery of bone marrow stromal cells (BMSCs) promotes neuronal regeneration and functional repair after stroke [127]. Furthermore, recent studies have shown that intranasal delivery of mitochondria has significant therapeutic effects on various neurological diseases, expanding the application scope of organelles in disease treatment [128,129] (Table 5).

#### 3.3.2. Research Progress in Exosome Intranasal Delivery

Exosomes are small extracellular vesicles (EVs) secreted by various cells that have multiple functions, including anti-apoptotic and anti-inflammatory effects. Compared to stem cells, exosomes are easier to store, have lower immunogenicity, and can be used as drug carriers [9]. Intranasal delivery of stem cell-derived exosomes has shown significant therapeutic effects on various CNS diseases, such as ischemic stroke, TBI, SCI, perinatal brain injury, cognitive impairments, PD, and autism [9,130]. For example, intranasal delivery of exosomes derived from human umbilical mesenchymal stem cells effectively alleviates brain injury, providing a new avenue for stroke treatment [131]. Additionally, intranasal delivery of stem cell-derived exosomes can significantly improve cognitive impairments after subarachnoid hemorrhage, reduce neuronal apoptosis and inflammation, and significantly alleviate symptoms of experimental autoimmune encephalomyelitis, reducing inflammatory cell infiltration and demyelination while enhancing BBB integrity [5]. Intranasal delivery of exosomes also reduces neuronal apoptosis and inhibits microglial inflammation [132].

### 3.4. Promising Formulations and Translational Potential

A variety of intranasal drug delivery systems demonstrate significant translational potential for CNS disorders, each with distinct advantages and limitations. Nanoparticle-based formulations offer high clinical potential due to their versatility, scalability, and compatibility with diverse drugs. Liposomes are effective in delivering both hydrophilic and lipophilic agents, though limited by short nasal residence time [133]. Polymeric nanoparticles (e.g., PLGA, chitosan) provide controlled release and targeted brain delivery, with some progressing to clinical trials [51,134]. Self-emulsifying drug delivery systems (SEDDS) are ideal for lipophilic CNS drugs, enhancing solubility and bypassing first-pass metabolism. They show reduced systemic toxicity but require particle size control (<200 nm) and more data on long-term nasal safety [135,136]. Hydrogel-based formulations are especially thermosensitive in situ gels; these platforms offer sustained release and improved nasal retention. Applications like berberine and self-healing hydrogels show enhanced therapeutic effects, though mucosal irritation and crosslinker biocompatibility remain challenges [137,138]. Exosome-derived systems leverage their innate CNS targeting and immune tolerance, and exosomes effectively deliver proteins and nucleic acids across the BBB. MSC-derived exosomes show promise in neuroregeneration, but production scalability and formulation stability are key hurdles [139,140,141]. Viral vectors (e.g., AAVs): Clinically validated for gene therapy in neurological diseases, these vectors offer efficient gene transfer and strong neuronal tropism [142,143]. Innovations like FUSIN improve delivery precision, but immunogenicity, payload limits, and regulatory complexity must be addressed [3,144]. Overall, these systems represent a robust and diverse toolkit for advancing non-invasive CNS therapies, with ongoing research focused on overcoming technical and safety-related challenges.

## 4. Advancements in Strategies to Enhance Intranasal Drug Delivery

### 4.1. Advances in Drug Delivery Devices for Intranasal Administration

To enhance the delivery of drugs into the brain, researchers explored various strategies, such as mechanical stimulation. New intranasal drug delivery devices play a key role in improving drug delivery efficiency within the intranasal cavity, enhancing drug distribution uniformity, increasing drug retention time on the intranasal mucosa, and improving patient compliance [145]. For example, spray devices that precisely control drug dosage and spray patterns ensure that drugs are accurately delivered to specific regions of the intranasal cavity, particularly the olfactory and respiratory regions, which are important for brain targeting. Furthermore, improvements in dry powder inhalers allow drugs to be more evenly dispersed as dry powders within the intranasal cavity, extending the drug’s action time in the nose [1,146]. New catheter technologies and spray device designs optimize drug delivery to the CNS, enhancing organ-specific drug concentration [147,148]. For example, optimizing the catheter insertion angle and depth with 3D printing ensures that drugs are accurately delivered to the olfactory regions, avoiding accidental inhalation or swallowing, providing a new method for highly accurate, reproducible, region-specific drug delivery [149]. New intranasal implant drug delivery systems have also been developed, using radioactive labeled risperidone, which allows non-invasive monitoring of drug release through MicroSPECT/CT imaging, offering a new path for CNS disease treatment [150].

Ultrasound-mediated intranasal drug delivery (FUSIN) technology enhances drug delivery efficiency through transcranial ultrasound, bypasses the BBB, and enables non-invasive brain drug delivery, demonstrating significant clinical application potential [151,152]. FUSIN technology bypasses the BBB via the nasal route, enhances drug delivery efficiency, and minimizes systemic exposure in major organs such as the heart, lungs, liver, and kidneys, showcasing its potential as a non-invasive gene therapy platform. For example, FUSIN technology can efficiently and safely deliver adeno-associated viruses (AAVs) to specific brain regions, offering advantages of low systemic exposure and non-invasiveness, highlighting its potential in gene therapy [153]. Furthermore, innovative intranasal drug delivery methods, such as magnetic stem cell micro-robots [154,155], spray delivery of neuroactive peptides [156], three types of spray devices for insulin delivery [157], and transcranial magnetic stimulation combined with magnetic nanoparticles [158], have further improved drug delivery efficiency.

### 4.2. Progress in Intranasal Drug Delivery with Nanotechnology

Nanotechnology has emerged as a promising strategy for intranasal drug delivery, offering enhanced retention time, mucosal penetrability, and uptake efficiency, particularly valuable for treating CNS disorders [11]. Among the various nanocarrier platforms, liposomal systems have significantly improved brain drug absorption [15,159], while polymer-based nanoparticles, nanoemulsions, and other formulations have enhanced the ability of drugs to cross the BBB [12,14,108,160,161]. Recent advances in nano- and microparticle-based delivery systems have shown considerable potential in enhancing the treatment of CNS diseases. Novel nanocomposites have been effective in managing memory deficits and neurological pathologies [162]. Additionally, hyaluronic acid-modified nanocarriers have enhanced brain targeting and cellular uptake, offering innovative treatment strategies for AD [163]. Notably, intranasal delivery of polymer-based nanoparticles has yielded superior therapeutic outcomes compared to traditional administration routes. For example, lamotrigine-loaded PLGA nanoparticles achieved higher brain concentrations and improved efficacy in animal models than oral delivery [164]. Similarly, phenytoin-loaded chitosan nanoparticles provided sustained release and elevated brain-specific drug levels in rats [165], while PEGylated nanoparticles encapsulating miR-132 successfully crossed the nasal-brain barrier and improved cognitive performance in AD mouse models [166]. Beyond small molecules, nanocarrier systems have facilitated the intranasal delivery of neuroactive peptides, oxytocin, insulin, and other biologics, thereby broadening their clinical application [167]. Emerging delivery platforms such as magnetic nanoparticles and plant-derived extracellular vesicles (EVs) have also been applied to treat epilepsy and gliomas, significantly enhancing drug targeting and immune responses [168,169,170]. Furthermore, advanced formulations such as PEG-modified chitosan-lipid nanocapsules and carbon nanotubes have exhibited notable potential in nasal drug delivery, contributing to improved therapeutic outcomes [171,172].

Several disease-specific applications further underscore the versatility of these systems. Zolmitriptan-loaded liposomes have shown effectiveness in migraine treatment [133]. Biodegradable nanoparticles delivering thyrotropin-releasing hormone demonstrated favorable safety profiles in preclinical models [173]. Research has also optimized drug delivery using various nanocarriers, including liposomes containing antioxidants, naringin nanocapsules, and rutin nanolamellar vesicles, significantly increasing brain drug concentrations and therapeutic efficacy, particularly in epilepsy, memory disorders, and neuroinflammation [174,175,176]. Moreover, studies have explored novel intranasal delivery methods using self-assembled nanoparticles, EVs, and miRNA nanoparticles, showing potential for improving neurodegenerative diseases, brain glioblastomas, and ischemic brain injury [177,178,179,180,181,182]. Additionally, spanlastic nanovesicles, prepared using film hydration and modified spray technologies, have enhanced the delivery efficiency of rasagiline mesylate to the brain [183]. Intranasal drug delivery of nanoemulsions has significant advantages in improving the efficiency of CNS drug delivery. Nanoemulsions can enhance the ability of drugs to penetrate the olfactory epithelium, thereby improving the local drug accessibility and brain delivery efficiency of CNS drugs [184]. For example, meloxicam nanoemulsions, zotepine microemulsions, and memantine nanoemulsions exhibit superiority in enhancing drug solubility, permeability, and organ-specific drug concentration, significantly enhancing therapeutic effects [185,186,187]. Moreover, butter and omega-3 fatty acid-rich fish oil, as permeation enhancers, significantly increase the ability of drugs to penetrate the nasal mucosa and enhance brain absorption [188]. Vitamin E-loaded naringin nanoemulsions and lactoferrin-modified huperzine A nanoemulsions demonstrate their potential in increasing drug concentration and reducing side effects [189,190,191].

In conclusion, intranasal drug delivery technology, in combination with nanotechnology and biomaterials, provides a new non-invasive therapeutic approach for CNS diseases, immunotherapy, cancer treatment, and other fields, demonstrating enormous clinical application potential [171,192,193].

### 4.3. Research Progress in Hydrogels for Intranasal Drug Delivery

Hydrogels have a promising application in intranasal drug delivery, effectively improving drug efficacy and safety and providing new approaches and methods for the development of intranasal drug delivery systems. For example, thermosensitive hydrogels delivering berberine for the treatment of depression not only bypass the BBB but also prolong the drug’s action time, significantly enhancing brain-specific drug concentration and improving antidepressant effects [194]. Self-assembled thermosensitive in situ hydrogels co-delivering berberine and evodiamine significantly improved the drug’s organ-specific drug concentration in intranasal drug delivery [195]. Loading resveratrol into sodium alginate nanogels successfully improved depressive behaviors in chronic stress rats, demonstrating strong antidepressant potential [196]. Hydrogels loaded with EVs for intranasal delivery can also treat myocardial ischemia–reperfusion injury, showing higher absorption efficiency and therapeutic potential, providing a new direction for treating myocardial infarction-related diseases [197,198,199]. Additionally, the AXT-NLC13-G4 system, based on nanolipid carriers and in situ gel technology, significantly improved the drug’s targeting to the brain and cognitive improvement effects [200]. Carboxymethyl chitosan and sodium hyaluronate hydrocolloid systems, with good wettability and rapid release properties, are suitable for insulin intranasal delivery [201]. Intranasal drug delivery technologies targeting specific brain regions, combined with self-healing supramolecular hydrogel systems, successfully increased drug concentrations in the brain while bypassing first-pass liver metabolism, providing effective treatment methods for neurological diseases [202]. Moreover, disulfide nanoparticle emulsified gels effectively treated glioblastoma through intranasal delivery, showing excellent brain targeting and safety [203]. A representative preclinical study utilized a chitosan hydrogel loaded with liposomal donepezil HCl, showing significantly enhanced brain targeting for the treatment of Alzheimer’s disease [204]. Similarly, a thermosensitive hydrogel highlighted the potential of temperature-sensitive hydrogels in facilitating direct drug delivery to the brain via the intranasal route [205]. In the field of peptide delivery, insulin-conjugated poly(N-vinyl pyrrolidone)-based nanogels can significantly enhance insulin concentration and biological activity in the brain [206]. These cases reflect the translational promise of hydrogel-based nasal systems, especially in CNS therapy, chronic disease management, and non-invasive alternatives to injections. Hydrogels offer significant advantages for intranasal drug delivery, including strong mucoadhesion, sustained release, and good biocompatibility, enabling effective brain targeting and bypassing the BBB. They have shown promising results in treating CNS disorders, such as depression and glioblastoma, especially when combined with nanoparticles or self-healing systems. However, limitations remain, including poor permeability for large biomolecules, sensitivity to nasal environmental conditions, and challenges in large-scale production. Compared to nanoparticles and SEDDS, hydrogels provide better safety and retention but moderate CNS delivery efficiency, making them ideal for small-molecule delivery and combinational strategies. These novel systems provide a non-invasive and efficient delivery route for the treatment of CNS diseases.

### 4.4. Research Progress on Permeation Enhancers in Intranasal Drug Delivery

Although intranasal drug delivery is non-invasive and efficient, many drugs are difficult to apply due to low bioavailability. Permeation enhancers play a crucial role in enhancing drug absorption and delivery efficiency [207]. Recent advancements in intranasal drug delivery have highlighted the pivotal role of permeation enhancers in improving the bioavailability of therapeutics, particularly for CNS disorders. Among these, alkylsaccharides such as dodecyl maltoside and tetradecyl maltoside have been extensively studied. These compounds transiently open tight junctions in the nasal epithelium, facilitating enhanced drug absorption without causing significant mucosal damage. Notably, dodecyl maltoside has been incorporated into several FDA-approved intranasal formulations, including diazepam for seizure clusters, nalmefene for opioid overdose, and sumatriptan for migraines, underscoring its clinical relevance [208]. For example, lauroylcholine chloride as a permeation enhancer significantly improved the drug’s delivery efficiency from the nasal cavity to the brain [209]. L-penetratin enhances direct drug entry into the brain through the olfactory mucosa, especially increasing hippocampal drug accumulation, providing a new therapeutic strategy for neurodegenerative diseases [210]. Additionally, a new platform of lactobacillus lactate was developed as a mucosal vaccine, demonstrating its broad-spectrum protection potential [211]. By improving the intranasal delivery formulations of ketamine, tunicamycin, and insulin, organ-specific drug concentration and brain delivery effects were significantly enhanced [212,213,214,215]. A novel PTD used as an absorption enhancer improved insulin delivery, providing a convenient solution for diabetes treatment [215]. Permeation enhancers offer significant advantages in intranasal drug delivery by improving mucosal absorption and overcoming the low bioavailability. Agents like lauroylcholine chloride and L-penetratin have been shown to enhance brain targeting, particularly increasing hippocampal accumulation, which is beneficial for neurodegenerative disease treatment [216,217]. Novel platforms, such as lactobacillus-based mucosal vaccines and PTD-assisted insulin delivery, further expand nasal delivery applications to immunotherapy and metabolic disorders. However, barriers remain, including potential mucosal irritation, variability in enhancer efficacy across individuals, and limited long-term safety data. Compared to traditional systems, permeation enhancers provide a practical, non-invasive means to boost drug bioavailability and brain targeting, especially when integrated into advanced formulations like nanoparticle or hydrogel-based systems [218,219]. These studies offer new insights for the application of intranasal drug delivery in the treatment of neurological diseases and other conditions (Table 6).

## 5. Challenges and Future Research Directions in Intranasal Drug Delivery

Intranasal drug delivery offers multiple advantages, including non-invasiveness, ease of administration, high patient compliance, and the ability to bypass the BBB, thus enabling direct drug transport to the CNS [8,14,228,229]. These benefits make it an attractive alternative to invasive routes like intravenous or intrathecal administration, particularly for chronic neurological conditions. However, intranasal drug delivery also faces several physiological and technical challenges. Drug absorption is often limited by inherent anatomical and physiological features such as the nasal epithelium’s barrier function, mucociliary clearance, enzymatic degradation, and variable solubility of drugs, which together affect both the extent and consistency of drug absorption [8]. Furthermore, local metabolic activity and rapid clearance mechanisms in the nasal cavity can significantly reduce drug bioavailability before it reaches the brain.

Formulation development remains a major hurdle, requiring careful optimization of drug stability, controlled release kinetics, mucoadhesive properties, and targeting capability to ensure effective CNS delivery [8]. Although intranasal drug delivery has demonstrated a favorable safety profile in non-human primate studies. It especially excels at increasing brain exposure while reducing systemic side effects. However, repeated administration over extended periods may lead to mucosal irritation and potential olfactory dysfunction, necessitating more rigorous investigation into its long-term safety in humans [230]. Drugs and excipients used in formulations, such as permeation enhancers, surfactants, or cationic polymers like chitosan, may disrupt nasal epithelial integrity, leading to neuronal inflammation, degeneration, or altered signaling. Repeated or high-dose exposure to neuroactive drugs can also result in excitotoxicity or oxidative stress in olfactory neurons and trigeminal fibers. Although many delivery systems demonstrate improved bioavailability, their safety on neuronal structures remains insufficiently characterized [1,231]. Therefore, it is essential for review studies to emphasize the need for preclinical and clinical evaluations of both drug- and excipient-induced neuronal toxicity to ensure safe and effective intranasal therapies.

Beyond physiological and formulation hurdles, intranasal drug delivery also faces regulatory and translational challenges. Currently, there is a lack of standardized FDA or EMA guidelines specifically tailored for CNS-targeted nasal delivery systems, and much of the regulatory evaluation relies on surrogate endpoints such as CSF levels rather than direct brain biodistribution [14,228]. Moreover, significant anatomical and physiological differences between animal models and humans contribute to discrepancies in clinical outcomes, as observed in trials like the modest translation of intranasal insulin efficacy from rodents to Alzheimer’s patients [229].

To overcome these limitations and fully realize the potential of intranasal drug delivery, future research should focus on the development of bioresponsive and smart formulations that adapt to the nasal environment, integration of AI and machine learning for personalized drug delivery optimization, establishment of predictive human-relevant in vitro and in silico models, and the design of scalable, cost-effective manufacturing platforms. In parallel, long-term clinical studies are needed to evaluate mucosal safety and efficacy across diverse populations [228,229].

This review identifies the following future research directions. First, it is essential to develop novel drug delivery systems, such as nanoparticles and gels, that effectively enhance drug absorption and minimize local side effects. Second, research should focus on individual differences in responses to intranasal drug delivery, aiming to develop personalized treatment plans to improve efficacy and reduce adverse effects. Additionally, long-acting, controlled-release systems are an important direction in intranasal drug delivery development. By controlling drug release, the duration of drug action can be extended, and the frequency of administration can be reduced. Furthermore, with the integration of artificial intelligence, in vitro experiments and computer simulations should be used to evaluate the behavior of intranasal drug delivery devices and formulations, combined with high-resolution imaging techniques and computational fluid dynamics simulations, to better understand the drug delivery process. Lastly, driven by single-cell technologies, efforts should be made to enhance the construction of the nasal-brain axis cell atlas, advancing intranasal drug delivery research from traditional pharmacology to the paradigm of precision medicine.

## 6. Conclusions

Intranasal drug delivery has emerged as a highly promising strategy for overcoming the limitations imposed by the BBB in the treatment of CNS diseases. As this review demonstrates, intranasal drug delivery methods provide direct, non-invasive access to the brain, enabling effective delivery of a wide range of therapeutics, including small molecules, biomacromolecules, nucleic acids, and cell-derived therapies. In addition, this method minimizes systemic exposure and enhances patient compliance. Mechanistically, both intracellular and extracellular transport mechanisms are involved, with formulation strategies such as nanoparticles, hydrogels, and permeation enhancers playing a central role in improving drug absorption, retention, and targeting efficiency. Significant progress has been made in optimizing delivery systems for various CNS disorders, including AD, PD, stroke, and brain tumors. Despite physiological and technical challenges, such as mucociliary clearance, local toxicity, and manufacturing complexities, the accumulating preclinical and clinical evidence supports the feasibility and translational potential of intranasal drug delivery. This review consolidates key advances and provides a comprehensive understanding of the mechanisms, applications, and challenges of this approach, offering a solid scientific foundation for its continued development in CNS therapeutics.

## Figures and Tables

**Table 1:** Types of drugs for intranasal drug delivery.

Drug Category	Specific Type	Relevant Examples
Chemical drugs	Solution dispersion	Solutions and suspensions
	Nanocarriers/polymers	Liposomes, nanoparticles, micelles, and hydrogels
Biomacromolecule drugs	Protein drugs	Recombinant proteins, antibodies, hormones, cytokines, and vaccines
	Nucleic acid drugs	DNA, RNA, and viruses
Cell-derived drugs	Cell-derived drugs	Stem cells, immune cells, and mitochondria
	Exosome-derived drugs	Exosomes from different sources

**Table 2:** Research progress of intranasal delivery of small molecule drugs.

Drug/ System Name	Application/Disease	Research Model	Key Findings	Ref.
Mixed Nanoparticle System	AD	Mouse Model	Rapid absorption, effective drug delivery to the brain, and high organ-specific drug concentration.	[51]
9-cis Retinoic Acid	AD	AD Transgenic Mouse	Reduces Aβ deposition and improves neuroinflammation and synaptic function.	[54]
Fluorobiprofene Microspheres/Soft Particles	AD	Rat Model	Intranasal powder delivery superior to solution, significant olfactory bulb concentration, and early intervention potential.	[64]
Lacosamide	Epilepsy	Mouse Model	Better pharmacokinetics compared to intravenous injection.	[52]
Opioids/Sedatives	Palliative Care	Clinical Study	Rapid onset, good patient tolerance, suitable for late-stage patients unable to take oral drugs.	[60]
Dihydrocodeine/Midazolam	Community Palliative Care (Pain/Agitation)	Clinical Practice	Easy to administer, improves patient comfort, and reduces medical delay.	[61]
Heparin	COVID-19 Prevention	Mouse and Human Trials	No significant toxicity and maintains effective concentration for 12 h.	[65]
Dexmedetomidine	Sedation for Extractions	Anxiety Patient Study	Onset in 30–45 min, lasts 60–75 min, no respiratory suppression, suitable for day surgeries.	[55]
MC4R Antagonist HS014	Trigeminal Neuralgia	Rat Model	Significant relief of hyperalgesia, upregulation of MC4R protein levels.	[56]
Ketamine	Post-Cesarean Pain Relief	Maternal RCT Study	Significantly reduces postoperative pain and morphine demand, and good tolerance.	[57]
Isocyanomethane	Epilepsy	Mouse Model	Rapidly increases seizure threshold and no motor/sedation side effects.	[53]
Lidocaine Spray	Post-Epidural Headache	Clinical Case	Non-invasive treatment, rapid symptom relief, replaces epidural blood patch.	[58]
Dexamethasone	Neuroinflammation (e.g., Stroke)	Mouse Model	Higher brain concentration, faster onset, and suitable for acute treatment.	[62]
Icariin-NGSTH System	Depression	Chronic Stress Rat Model	Faster antidepressant effect and organ-specific drug concentration significantly better than oral administration.	[63]
Naltrexone	Opioid Side Effects	Rodent Model	Alleviates gastrointestinal and central side effects without affecting analgesic effect.	[59]
Chlorpyrifos	Neurotoxicity	Adult Male Mouse	High doses cause memory impairment, anxiety, and brain oxidative stress.	[65]

**Table 3:** Research progress in intranasal delivery of biologic drugs.

Drug/ System Name	Application/ Disease	Research Model	Key Findings	Ref.
NGF	TBI	Clinical Trial	Bypasses the BBB to directly affect brain tissue, reducing systemic side effects.	[67,70]
Leukemia Inhibitory Factor (LIF)	Mild TBI (mTBI) in Children	CD1 Mice	Alleviates glial proliferation and axonal damage, improves sensory-motor function, and has no side effects.	[71]
Insulin	AD	Rat Model	Intranasal insulin rapidly distributes to the brain, improves cognitive function, and optimized formulation reduces systemic side effects.	[80]
Insulin	AD	Multicenter Clinical Trial	Improves memory performance in patients with mild cognitive impairment or AD.	[77]
Insulin (Nanocarrier Technology)	CNS Diseases	Rat Model	Enhances delivery efficiency with nanotechnology, bypasses the BBB through olfactory or trigeminal nerves, and significantly improves cognitive function.	[74,75]
Insulin	PD	Rat Model	Directly targets the brain and minimizes systemic side effects.	[78]
IGF-1	Brain Ischemia	Rat Model	Reduces neural damage and inflammation and bypasses the BBB to directly affect the brain.	[72]
Oxytocin Intranasal Spray TTA-121	ASD	Rabbit Model	Significantly higher brain-specific drug concentration compared to Syntocinon, higher concentrations in the prefrontal cortex and cuneus.	[84]
Insulin (PTD-Modified Formulation)	Diabetes	Rat Experiment	Enhances absorption with Protein Transduction Domain (PTD) and optimizes intranasal delivery formulation to improve efficacy.	[79]
Bicyclic Peptide OL-CTOP	Morphine Side Effect Antagonism	Mouse	Intranasal delivery effectively antagonizes morphine’s analgesic and respiratory suppression side effects, demonstrating potential for brain-targeted delivery.	[87]

**Table 4:** Research progress in intranasal delivery of nucleic acid drugs.

Drug/System Name	Application/Disease	Research Model	Key Findings	References
Self-Assembled Antagomir-21/RAP Nanoparticles	Glioblastoma	Mouse Model	Enhanced efficacy with a non-toxic carrier, effectively inhibiting tumor growth.	[113]
ApoA-I Nanodisk-Loaded ASO (Antisense Oligonucleotide)	Huntington’s Disease	HD Mouse Model	Single intranasal delivery significantly reduces mutated Huntington protein (mHTT) levels in the striatum and cortex.	[114]
Glycerol Chitosan-DNA Complex (GCP/GCPH)	Neurological Diseases (e.g., AD)	Mouse Model	GCP targets gene delivery to the cerebral cortex; GCPH (hyaluronidase-coated) enhances brain distribution.	[115]
MSC-Exo-loaded PTEN siRNA (Mesenchymal Stem Cell Exosomes)	Complete SCI	Animal Model	Non-invasive intranasal delivery promotes functional recovery after SCI.	[116]

**Table 5:** Research progress in intranasal delivery of cell therapy drugs.

Drug/System Name	Application/ Disease	Research Model	Key Findings	Ref.
Mitochondria	Chemotherapy-induced Cognitive Deficits	Mouse Model	Intranasal delivery of mitochondrial-targeted compounds provides neuroprotective effects.	[128,129]
Human Olfactory Mucosal Progenitor Cells (OMPCs)	Brain Injury	Rat Diffuse Axonal Injury Model	OMPCs migrate to the vicinity of damaged neurons and axons via intranasal delivery, supporting non-invasive stem cell therapy.	[120]
Human Neural Stem Cells (hNSCs)	AD	AD Mouse Model	hNSCs survive and differentiate into neurons, reducing β-amyloid plaque accumulation and synapse loss, improving cognitive function.	[122]
Bone Marrow-Derived Mesenchymal Stem Cells (BMSCs)	PD	PD Mouse Model	Pre-treated BMSCs enhance efficacy, improving motor function and reducing dopaminergic neuron loss.	[123]
Human Umbilical Cord-Derived Mesenchymal Stromal Cells (MSCs)	Bronchopulmonary Dysplasia (BPD)	Experimental BPD Model	Intranasal delivery of MSCs repairs lung damage caused by BPD with simple methods and clinical potential.	[125]
Deciduous Dental Pulp Stem Cells (DPSCs)	PD	MPTP-induced PD Mouse	DPSCs improve motor coordination and olfactory function, reducing dopaminergic neuron degeneration.	[124]
Delayed Repeated Intranasal Delivery of Bone Marrow Stromal Cells	Ischemic Stroke	Mouse Stroke Model	Delayed repeated intranasal delivery promotes regeneration and functional recovery after stroke.	[127]

**Table 6:** Advantages and limitations of nanocarrier types in intranasal drug delivery systems.

Nanocarrier Type	Advantages	Limitations	Ref.
Liposomes	High biocompatibility; good drug loading for hydrophilic drugs.	Rapid clearance; low stability.	[220]
Polymeric Nanoparticles (PLGA, Chitosan)	High drug loading; sustained release; improved stability; versatile surface modification.	Potential toxicity; difficulty in large-scale production.	[221]
Nanoemulsions	Enhanced solubility and permeability of lipophilic drugs.	Thermodynamic instability, need stabilizers.	[222]
Magnetic Nanoparticles	Magnetic targeting, imaging compatibility.	Potential safety concerns, complex formulation.	[223]
PEGylated Nanoparticles	Extended circulation time, improved CNS penetration.	Expensive, potential immune response.	[224]
Exosomes	Endogenous origin; high biocompatibility; excellent penetration across biological barriers.	Low production yield; difficulty in drug loading; high cost.	[225]
Plant-derived Extracellular Vesicles (EVs)	Natural origin; low toxicity; immune modulation potential.	Scalability; batch variability.	[226]
Hydrogel-nanoparticle hybrids	Mucoadhesion; biocompatibility; prolonged residence time.	Formulation optimization challenges; drug release control.	[227]

## Abbreviations

AAVs	Adeno-associated viruses
AD	Alzheimer’s disease
ASO	Antisense oligonucleotide
BBB	Blood–brain barrier
BMSCs	Bone marrow-derived mesenchymal stem cells
BPD	Bronchopulmonary dysplasia
CSF	Cerebrospinal fluid
CNS	Central nervous system
DPSCs	Deciduous dental pulp stem cells
EVs	Extracellular vesicles
FUSIN	Focused ultrasound-mediated nasal drug delivery
GLP-1	Glucagon-like peptide-1
IGF-1	Insulin-like growth factor 1
hNSCs	Human neural stem cells
LIF	Leukemia inhibitory factor
MSC	Mesenchymal stem cell
NGF	Nerve growth factor
OMPCs	Olfactory mucosal progenitor cells
PD	Parkinson’s disease
PEG	Polyethylene glycol
PTD	Protein transduction domain
SAD	Social anxiety disorder
SCI	Spinal cord injury
TBI	Traumatic brain injury
ZO-1	Zonula occludens-1
